# Successful Surgical Management of Traumatic Carotid‐Jugular Fistula in an Afghan Patient: A Case Report

**DOI:** 10.1002/ccr3.70816

**Published:** 2025-08-22

**Authors:** Ramin Rouhafza, Mohammad Manaviat, Hamidreza Ghasemirad, Mobin Fallah Tafti, Seyed Masoud Hosseini

**Affiliations:** ^1^ School of Medicine Shahid Sadoughi University of Medical Sciences Yazd Iran; ^2^ Students' Research and Technology Committee Shahid Sadoughi University of Medical Sciences Yazd Iran; ^3^ Student Research Committee Shiraz University of Medical Sciences Shiraz Iran; ^4^ Department of General Surgery Shahid Sadoughi University of Medical Sciences Yazd Iran

**Keywords:** carotid‐jugular fistula, case report, open surgical repair, traumatic arteriovenous fistula, vascular trauma

## Abstract

A 23‐year‐old Afghan man presented with a traumatic carotid‐jugular fistula (CJF) and a right common carotid pseudoaneurysm following a gunshot wound. Persistent neck pain and pulsation led to imaging, which revealed an aneurysmal dilation and fistulous connection. He underwent successful open surgical repair, which included resection of the aneurysm and reconstruction of the affected vessels. Postoperative recovery was marked by the resolution of symptoms and normal function. This case underscores the effectiveness of open surgery for complex CJFs.

**Summary**

- Open surgical repair is a viable and effective approach for managing traumatic CJFs, providing complete resolution in complex cases where endovascular methods may not be feasible, particularly in resource‐limited settings.

## 1 Introduction

Arteriovenous fistulas (AVF) may result from an arterial injury and appear grossly as pathologic connections between an artery and a vein, causing reduced blood flow to the distal tissues relative to the flow through the AVF [1]. Traumatic carotid‐jugular fistulas (CJF) have only been reported in 4% to 7% of all cases of traumatic AVFs [1, 2, 3]. If Runtreated, these rare injuries can result in congestive heart failure, atrial fibrillation, cerebral ischemia, embolization, and bleeding [4, 5].

The current management options for CJFs include open surgery, which, despite its higher complexity, provides direct access and the possibility of complete repair, making it a suitable choice in certain complex cases, and endovascular surgery as a less invasive method indicated in some cases [1, 5, 6]. We reported the successful open surgery repair of a right common carotid pseudoaneurysm in a 23‐year‐old Afghan man who presented with CJF after a gunshot wound. As far as we know, this case was the first case report of traumatic CJF surgery conducted in Iran and also the first CJF case in the Afghan people. This case not only adds to the limited existing literature on traumatic CJFs but also demonstrates the viability of open surgical intervention in resource‐limited settings, underscoring its significance in clinical practice.

## 2 Case Presentation

### 2.1 History and Examination

A 23‐year‐old Afghan man was admitted to the general surgery department with a 6‐month history of throbbing pain and pulsation in the right side of his neck. Nine months prior to admission, he experienced a penetrating trauma from a gunshot wound to the right lateral side of his neck, for which he underwent bullet removal surgery immediately.

On admission, the patient was hemodynamically stable, including a pulse rate of 83, a respiratory rate of 16 breaths/min, blood pressure of 128/79 mmHg, a temperature of 36.8, and oxygen saturation of 99%. On physical examination, the patient was awake and alert to time, place, and person.

Auscultation over the neck identified a significant venous systolic thrill on the right lateral side. Cardiovascular examinations revealed normal S1 and S2 with full and symmetric peripheral pulses. Neurological examinations, including sensation, strength, and reflexes, were absolutely normal.

### 2.2 Differential Diagnosis, Investigations, and Treatments

Laboratory tests, including levels of complete blood count, electrolytes, urea, and creatinine, were within normal limits.

**FIGURE 1:**
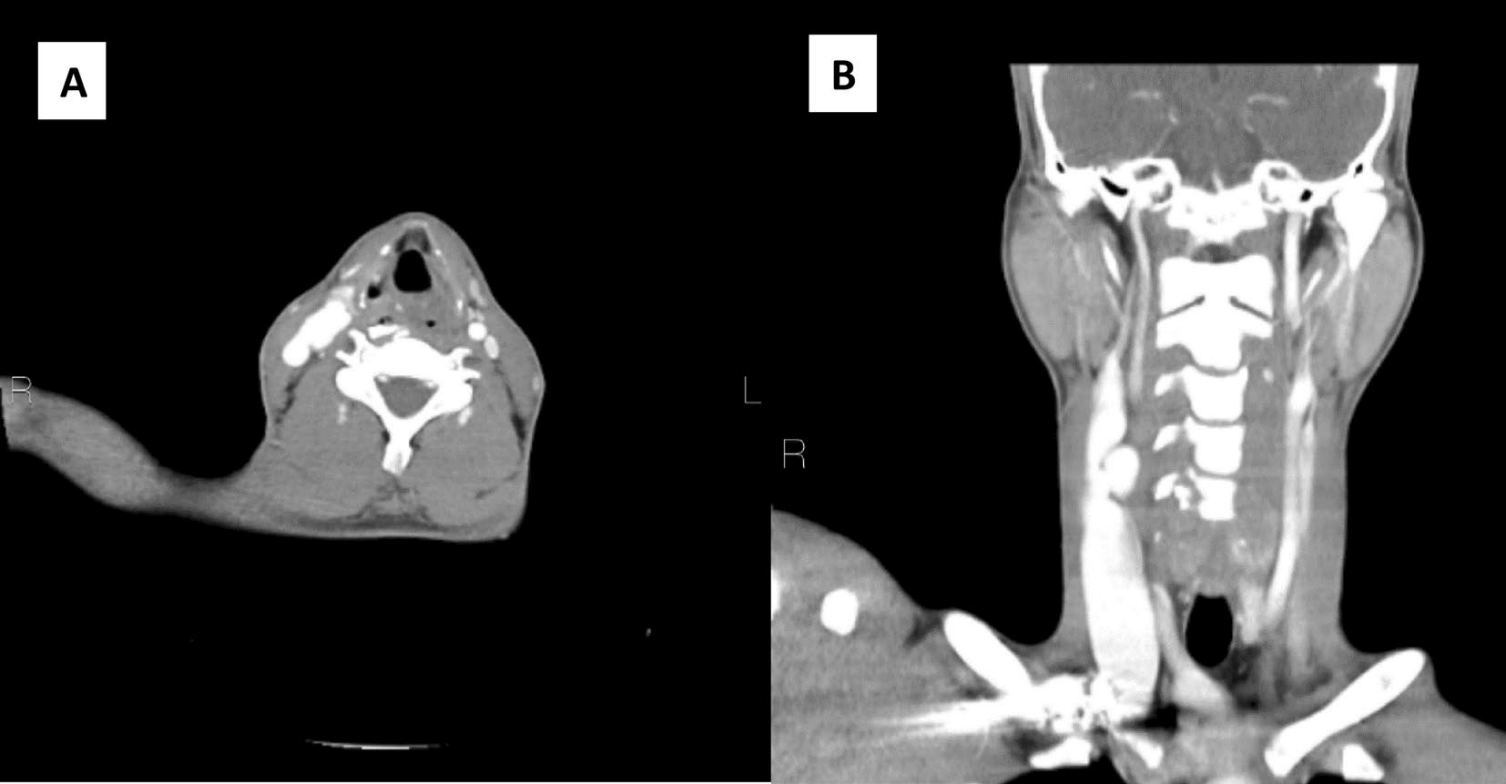
Spiral CT‐Scan of neck with IV Contrast (A: Axial View—B: Coronal View). Aneurysmal dilation of the distal CCA (15*14 mm) with fistula to the right Jugular Vein (AVF).

Contrast‐enhanced spiral Computed‐Tomography of the neck demonstrated aneurysmal dilation of the distal common carotid artery (15*14 mm) with an arteriovenous fistula involving the right internal jugular vein (Figure 1).

**FIGURE 2:**
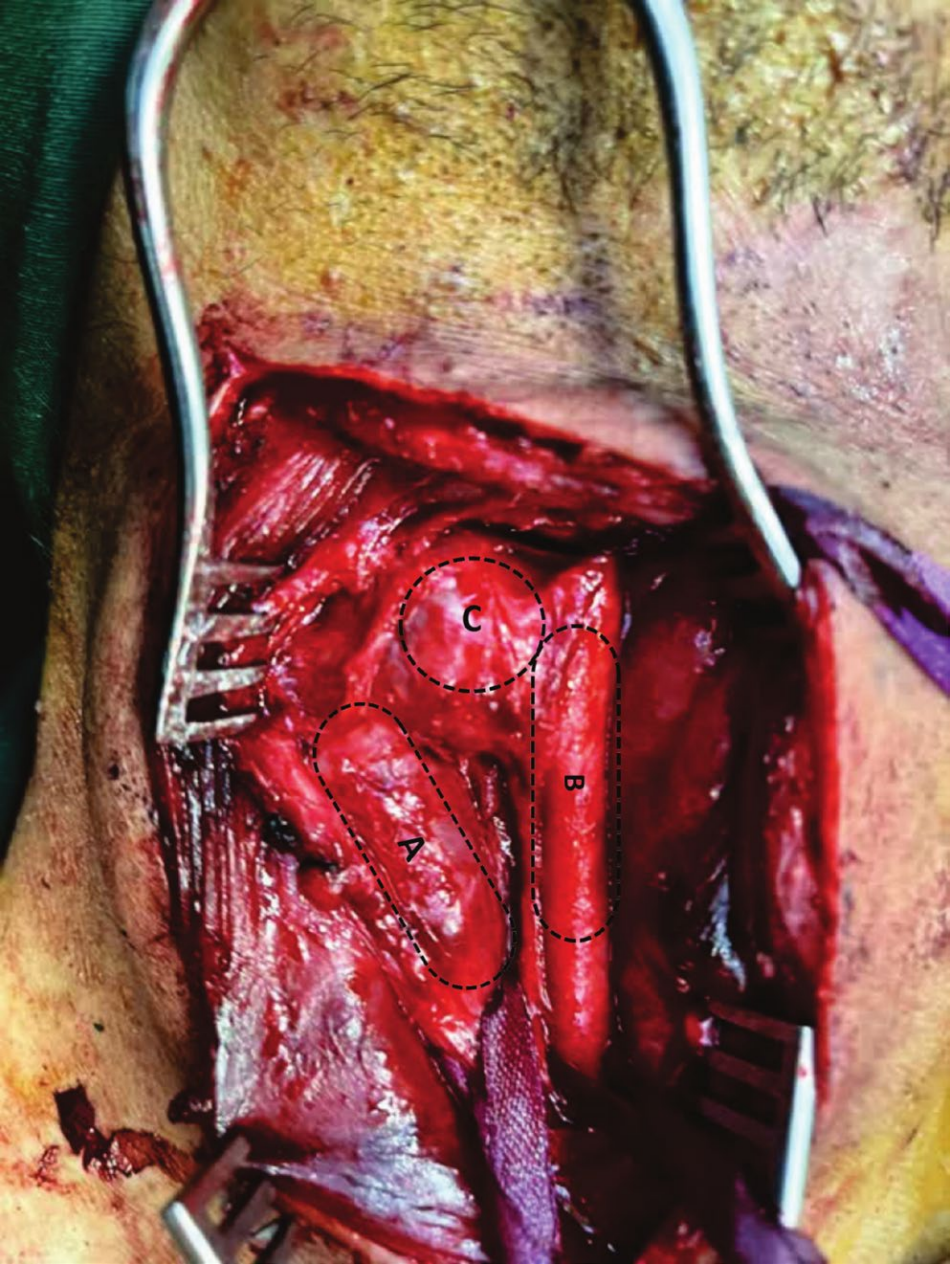
Fistula zone before surgery: (A) Internal jugular vein (B) Common Carotid Artery (C) carotid‐jugular fistula.

**FIGURE 3:**
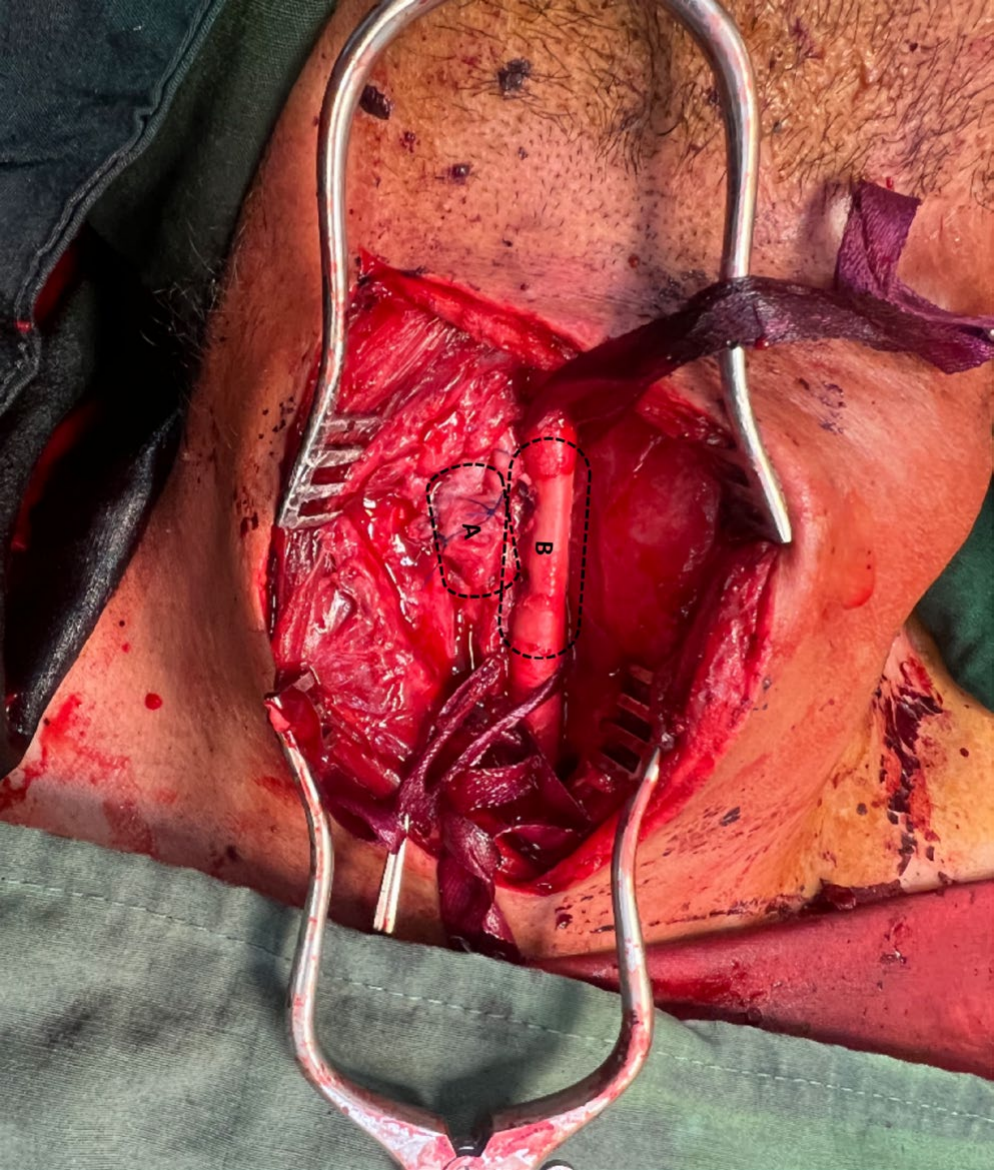
Fistula zone after surgery: (A) Repaired Internal jugular Vein (B) Repaired Common carotid Artery.

The patient was prepared for open vascular surgery. After induction of general anesthesia, skin and subcutaneous tissue were incised anteriorly to the sternocleidomastoid muscle (SCM) in a semi‐extension and left‐rotation position. Then, the skin and subcutaneous tissue over the fistula site were opened. Strap muscles were opened, and exploration of the right common carotid artery and internal jugular vein was performed. The presentation of the fistula was shown in Figure 2. The common carotid artery was controlled proximally. The artery was aneurysmal at the fistula site. The jugular vein was controlled from the proximal and distal sites of the AVF. Five thousand units of heparin were injected. After 3 min, the artery and internal jugular vein were clamped proximally and distally at the fistula site. Then, the internal jugular vein was gently separated from the carotid artery. The distal and proximal segments of the common carotid artery were clamped for 5 min; subsequently, the aortic clamp was removed elliptically at the aneurysmal site for reduction of the risk of embolization, and arterial repair was performed using 6–0 Prolene sutures. Subsequently, a leak test was performed; blood flow was pulsatile and adequate distal to the site of aneurysm repair. The fistula was removed elliptically at the aneurysmal site. The aneurysm was repaired. A leak test was performed, and distal pulsatile flow was adequate. Then, the internal jugular vein was mobilized from the surrounding tissues and repaired at the fistula site using Prolene 6–0 sutures. The SCM was released, and it was placed between the repaired artery and vein. Hemostasis was achieved. A Hemovac drain was inserted. The surgery site wound was closed with subcuticular Monocryl sutures (Figure 3 represents the surgical site after the repairment).

### 2.3 Outcome and Follow‐Up

After the surgery, the patient went on to complete vascular and neurological examinations. The patient's sensation, movement, and muscle strength were normal, and the distal pulses were full and similar to each other. The vital signs were normal and stable. After two days, he was discharged with improved signs and symptoms.

## 3 Discussion

CJFs represent complex vascular injuries that require skilled diagnostic and therapeutic strategies. These rare and potentially life‐threatening conditions demand comprehensive medical evaluation and precise intervention to prevent severe complications such as cerebral ischemia, systemic embolization, hemodynamic disruptions, and heart failure [7].

In our case, the CJF originated from a penetrating gunshot wound, highlighting the intricate nature of traumatic vascular injuries. The diagnostic process relied on critical clinical findings, including a venous systolic thrill and radiographic evidence of a pseudoaneurysm, which are essential for accurate assessment and subsequent management [8].

The selection of treatment methods requires careful consideration of multiple factors. Open surgical repair emerged as the preferred approach, offering significant advantages over alternative interventions. However, certain absolute and relative contraindications must be thoroughly evaluated before proceeding with surgical intervention.

Absolute contraindications for open surgical repair include severe systemic coagulopathy, uncontrolled cardiovascular instability, and advanced multiorgan failure. Relative contraindications encompass age with multiple comorbidities, a compromised immune system, recent myocardial infarction, inadequate nutritional status, and tracheostomy [9].

Endovascular treatments present their own set of limitations, including challenges with complex anatomical vascular configurations, small vessel diameter, high embolic risk, and potential long‐term medication compliance issues. The chosen surgical approach provided several critical benefits: direct visualization of vascular pathology, immediate structural reconstruction, comprehensive control of underlying tissue damage, and lower long‐term embolic complication risks; endovascular treatments, while less invasive, demonstrate significant drawbacks: prolonged anticoagulation requirements, higher risk of repeated interventions, and increased potential for long‐term complications [10].

Critical factors influencing treatment selection included the patient's young age, specific anatomical injury characteristics, low socioeconomic situation affecting follow‐up, potential for comprehensive rehabilitation, and individual risk profile.

The successful management of traumatic CJFs through open surgical repair, as demonstrated in our case report, aligns with findings from several studies in the literature.

For instance, Alonso‐Argüeso et al. emphasized the effectiveness of endovascular treatment for complex CJFs, but they also acknowledged the limitations of this approach in specific cases where direct surgical intervention is necessary. Their research highlights that while endovascular methods can be less invasive, they may not provide the comprehensive repair needed for complex vascular injuries, particularly in resource‐limited settings [1].

Similarly, Sinha et al. reported a case of a traumatic common carotid‐internal jugular arteriovenous fistula that required surgical intervention due to life‐threatening complications, reinforcing the notion that open surgery remains a viable option when faced with significant anatomical challenges [2].

Furthermore, Góes et al. provided insight into surgical repair techniques for traumatic CJFs, noting that direct access allows for thorough exploration and repair of the affected vessels. This perspective is echoed in our case, where open surgical repair not only addressed the immediate vascular injury but also ensured long‐term stability and function [2].

Overall, these studies collectively underscore the importance of individualized treatment approaches based on patient‐specific factors and the complexity of the injury, supporting the efficacy of open surgical repair as a critical option in managing traumatic CJFs.

## 4 Conclusion

This case highlights the successful open surgical repair of a traumatic CJF in a young Afghan patient with limited financial resources. The patient's socioeconomic constraints and the lack of access to endovascular interventions in a resource‐limited setting necessitated a cost‐effective, definitive surgical approach. Open repair not only resolved symptoms but also minimized long‐term costs and follow‐up burdens, which are critical for patients facing economic hardship. The outcome underscores the importance of tailoring treatment strategies to local resource availability and patient‐specific socioeconomic factors.

## Author Contributions

**Ramin Rouhafza:** conceptualization, data curation, methodology. **Mohammad Manaviat:** methodology, resources, writing – original draft, writing – review and editing. **Hamidreza Ghasemirad:** conceptualization, data curation, investigation, writing – original draft, writing – review and editing. **Mobin Fallah Tafti:** investigation, methodology, writing – original draft, writing – review and editing. **Seyed Masoud Hosseini:** conceptualization, data curation, investigation, methodology, writing – original draft, writing – review and editing.

## Ethics Statement

Informed consent was obtained from the patient; the Helsinki 1964 Declaration and its later amendments were observed.

## Consent

The patient gave written informed consent to publish this report in accordance with the journal's patient consent policy.

## Conflicts of Interest

The authors declare no conflicts of interest.

## Data Availability

Data sharing is not applicable to this article as no new data were created or analyzed in this study.

## References

[1] G. Alonso‐Argüeso , A. Rodriguez‐Morata , B. Vera‐Arroyo , M. J. Lara‐Villoslada , and R. Gomez‐Medialdea , “Endovascular Treatment of Complex Carotid‐Jugular Fistula,” Vascular and Endovascular Surgery 50, no. 8 (2016): 566–570. 27852880 10.1177/1538574416675675

[2] V. K. Sinha , A. Yaduvanshi , V. Kataria , and M. Nair , “Traumatic Common Carotid–Internal Jugular Arteriovenous Fistula Manifesting as Life‐Threatening Epistaxis,” JACC Case Reports 1, no. 4 (2019): 576–578. 34316882 10.1016/j.jaccas.2019.08.023 PMC8289081

[3] M. A. El‐Shahawy and H. Khilnani , “Carotid‐Jugular Arteriovenous Fistula: A Complication of Temporary Hemodialysis Catheter,” American Journal of Nephrology 15, no. 4 (2008): 332–336. 10.1159/000168859 7573193

[4] N. Ezemba , E. E. Ekpe , H. A. Ezike , and C. H. Anyanwu , “Traumatic Common Carotid‐Jugular Fistula: Report of 2 Cases,” Texas Heart Institute Journal 33, no. 1 (2006): 81–83. 16572879 PMC1413591

[5] A. M. O. Góes , S. A. H. Jeha , D. V. Dias , and J. M. T. Ferreira , “Surgical Repair of a Traumatic Carotid‐Jugular Arteriovenous Fistula,” Jornal Vascular Brasileiro 19 (2020): e20200008. 34211512 10.1590/1677-5449.200008 PMC8218008

[6] M. Massara , D. Barillà , G. De Caridi , et al., “An Hybrid 2‐Stage Technique to Treat a Post‐Traumatic Internal Carotid‐Jugular Fistula,” Annals of Vascular Surgery 38 (2017): 315. 10.1016/j.avsg.2016.05.094 27522967

[7] M. R. Islam , A. Ansari , A. Rahman , et al., “The Perplexing Postsurgical Complication of Carotid‐Jugular Fistula: A Bitter Experience,” Surgical Neurology International 13 (2022): 2. 35127202 10.25259/SNI_967_2021 PMC8813626

[8] A. Dammak , H. Ben Jmaà , S. Hadhri , et al., “Post‐Traumatic Carotido‐Jugular Fistula: Case Report and Review of the Literature,” Journal De Médecine Vasculaire 42, no. 6 (2017): 388–391. 29203046 10.1016/j.jdmv.2017.09.005

[9] F. Johnson , A. M. H. Ho , R. Allard , and G. B. Mizubuti , “Relative Positions of the Right Internal Jugular Vein and the Right Common Carotid Artery,” Postgraduate Medical Journal 98, no. e1 (2022): e16–e17. 10.1136/postgradmedj-2020-138125 37066555

[10] E. D. Dillavou , S. C. Muluk , and M. S. Makaroun , “Improving Aneurysm‐Related Outcomes: Nationwide Benefits of Endovascular Repair,” Journal of Vascular Surgery 43, no. 3 (2006): 446–512. 16520153 10.1016/j.jvs.2005.11.017

